# Transient Calcium and Dopamine Increase PKA Activity and DARPP-32 Phosphorylation

**DOI:** 10.1371/journal.pcbi.0020119

**Published:** 2006-09-08

**Authors:** Maria Lindskog, MyungSook Kim, Martin A Wikström, Kim T Blackwell, Jeanette Hellgren Kotaleski

**Affiliations:** 1 School of Computer Science and Communication, Royal Institute of Technology, Stockholm, Sweden; 2 School of Computational Sciences, George Mason University, Fairfax, Virginia, United States of America; 3 The Krasnow Institute for Advanced Study, George Mason University, Fairfax, Virginia, United States of America; 4 Department of Neuroscience, Karolinska Institutet, Stockholm, Sweden; University College London, United Kingdom

## Abstract

Reinforcement learning theorizes that strengthening of synaptic connections in medium spiny neurons of the striatum occurs when glutamatergic input (from cortex) and dopaminergic input (from substantia nigra) are received simultaneously. Subsequent to learning, medium spiny neurons with strengthened synapses are more likely to fire in response to cortical input alone. This synaptic plasticity is produced by phosphorylation of AMPA receptors, caused by phosphorylation of various signalling molecules. A key signalling molecule is the phosphoprotein DARPP-32, highly expressed in striatal medium spiny neurons. DARPP-32 is regulated by several neurotransmitters through a complex network of intracellular signalling pathways involving cAMP (increased through dopamine stimulation) and calcium (increased through glutamate stimulation). Since DARPP-32 controls several kinases and phosphatases involved in striatal synaptic plasticity, understanding the interactions between cAMP and calcium, in particular the effect of transient stimuli on DARPP-32 phosphorylation, has major implications for understanding reinforcement learning. We developed a computer model of the biochemical reaction pathways involved in the phosphorylation of DARPP-32 on Thr34 and Thr75. Ordinary differential equations describing the biochemical reactions were implemented in a single compartment model using the software XPPAUT. Reaction rate constants were obtained from the biochemical literature. The first set of simulations using sustained elevations of dopamine and calcium produced phosphorylation levels of DARPP-32 similar to that measured experimentally, thereby validating the model. The second set of simulations, using the validated model, showed that transient dopamine elevations increased the phosphorylation of Thr34 as expected, but transient calcium elevations also increased the phosphorylation of Thr34, contrary to what is believed. When transient calcium and dopamine stimuli were paired, PKA activation and Thr34 phosphorylation increased compared with dopamine alone. This result, which is robust to variation in model parameters, supports reinforcement learning theories in which activity-dependent long-term synaptic plasticity requires paired glutamate and dopamine inputs.

## Synopsis

Reinforcement learning, based on the association of a stimulus-triggered movement with a reward, involves changes in connection strength between neurons. Memory storage occurs in the striatum, the input stage of the basal ganglia, when a stimulus or movement signal originating from the cortex and a reward signal originating from the midbrain reach the target striatal cells together. Repetitive pairing of these two signals strengthens the connection between cortical and striatal cells. The strengthening of the connections is caused by activation of biochemical signalling pathways inside the striatal cells. These intracellular signalling pathways are explored in a quantitative computational model describing the biochemical pathways important for reinforcement learning. Lindskog et al.'s study shows that when brief reward and stimuli signals are paired, a stronger response in the intracellular signalling occurs compared with the situation when each signal is given alone. This result illustrates mechanisms whereby paired stimuli, but not unpaired stimuli, can cause learning. Furthermore, the model predicts that the biochemical responses are different after brief stimulation as compared with prolonged stimulation. This result highlights the difficulties in predicting the nonlinear interactions within signalling cascades based on prolonged stimulations, which often are used in biochemical experiments.

## Introduction

The basal ganglia play an important role in reinforcement learning, in which an animal learns that performing a particular action in response to a neutral (e.g., visual) stimulus will be rewarded [1,2]. In vivo recordings during learning reveal that the reward elicits an increase in dopamine neuron firing [3], and an increase in dopamine release in the striatum [4,5], whereas the visual stimulus and the motor action produce cortical activity, which is transmitted to the striatum as an increase in glutamate release. Thus, during reinforcement learning, the medium spiny neurons of the striatum receive paired glutamate and dopamine input. Numerous studies show that glutamate input from cortex combined with dopamine produces a persistent increase in the size of the glutamatergic EPSC of medium spiny neurons, which is known as long-term potentiation (LTP). These observations support the hypothesis that synaptic plasticity of medium spiny neurons underlies reinforcement learning: the cortico–striatal synapses that are active simultaneously with dopamine input are potentiated during reinforcement learning [6,7]. Equally important to theoretical models of reinforcement learning is that synaptic potentiation does not occur in response to dopamine or glutamate signals alone [8]. Nonetheless, the subcellular mechanisms within medium spiny neurons underlying the requirement for paired stimuli are not completely understood.

Synaptic plasticity is controlled by the state of phosphorylation of various components in the intracellular signalling network [9,10]. Phosphorylation of Ser 845 on the GluR1 subunit by cAMP-dependent kinase (PKA) increases insertion of AMPA receptors into the membrane, whereas dephosphorylation by protein phosphatase 1 (PP1) has the opposite effect [11,12]. In the medium spiny neurons of the striatum, the balance between PKA and PP1 is heavily regulated by the phosphoprotein DARPP-32 (dopamine- and cAMP-regulated phosphoprotein of 32 kDa) [13].

The activity of DARPP-32 is regulated mainly by two phosphorylation sites: threonine (Thr) 34 and Thr 75 (rat sequence). When phosphorylated at Thr34, DARPP-32 is a potent inhibitor of PP1 [14], whereas when phosphorylated at Thr75 DARPP-32 inhibits protein kinase A (PKA) [15]. The state of phosphorylation of DARPP-32 has been shown to be regulated by dopamine and several other neurotransmitters, including glutamate [16,17], adenosine [18,19], and opioids [20]. Thr34 is phosphorylated by PKA and dephosphorylated by protein phosphatase 2B (PP2B or calcineurin) [16,21] whereas Thr75 is phosphorylated by the cyclin-dependent kinase 5 (cdk5) and dephosphorylated mainly by PP2A [15,17]. A vast amount of data regarding the regulation of phosphorylation of DARPP-32, as well as its effect on other intracellular proteins, has been generated in the last few years (for review see [13]). Nonetheless, it is not evident how co-activation of multiple neurotransmitters modulates the signalling network.

To gain a better understanding of the mechanisms regulating DARPP-32 phosphorylation and its effect on kinase and phosphatase activity and subsequent plasticity, we developed a computer model of this signalling network, based on the available experimental data. The model allows us to investigate the effect of transient glutamate and dopamine stimuli on levels of signalling molecules and the phosphorylation of DARPP-32; and to identify critical reactions within the network. We find that transient stimulation produces different enzyme activation compared with the prolonged treatments typically used in biochemical experiments. For example, short pulses of calcium influx increase DARPP-32 phosphorylation at Thr34 compared with the decrease seen with prolonged stimulation. Another finding is that the feedback loop PKA–PP2A–phosphoThr75 does not exclusively reinforce the PKA pathway, as previously hypothesized, but at times acts as a sink for catalytic PKA, dampening the stimulatory effect of dopamine.

## Results

### Model Verification: Response to Steady-State Inputs

**Figure 1:**
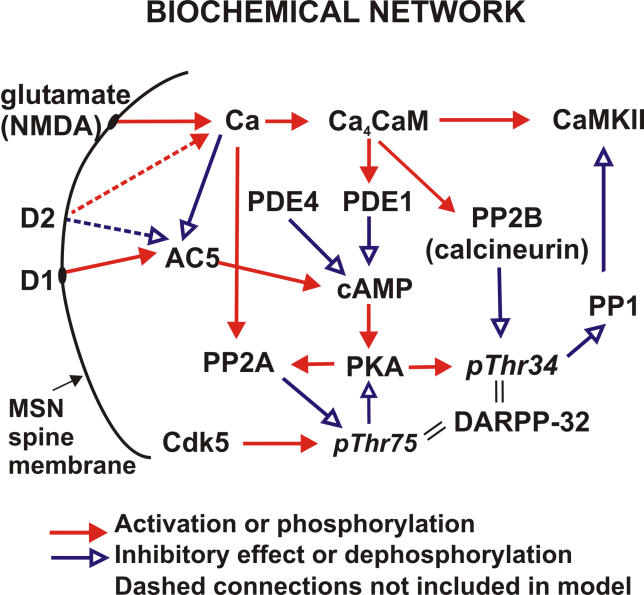
Second Messenger Pathways Involved in the Phosphorylation of DARPP-32 on Thr34 and Thr75 in Medium Spiny Projection Neurons A calcium elevation produced by glutamate leads to calcium binding to CaM (Ca_4_CaM), which activates both CaMKII and PP2B, the latter dephosphorylating DARPP-32 on Thr34. Stimulation of the dopamine D_1_ receptor activates the PKA cascade via AC5 and cAMP formation. PKA in its turn increases phosphorylation of DARPP-32 on Thr34, which then inhibits PP1.

The model, illustrated in Figure 1, consists of ordinary differential equations describing mass action kinetics of biochemical reactions known to occur in striatal medium spiny neurons. Tables 1–4 list these reactions, as well as their rate constants and the quantities of molecules used in the model. Equations are derived assuming all reactions are in a single compartment (i.e., a spine).

**Table 1:**
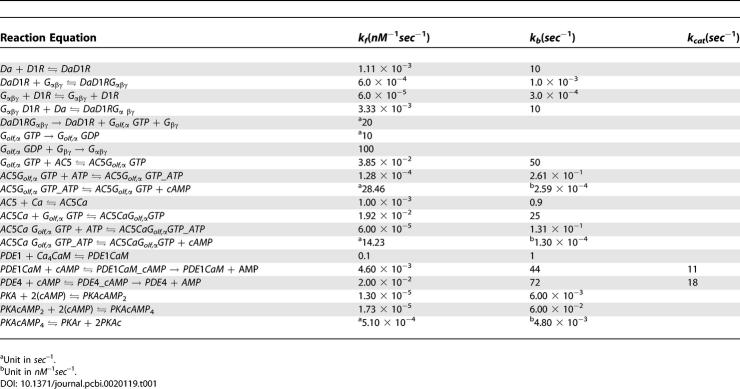
Reactions and Rate Constants of Dopamine–PKA Pathway

**Table 2:**
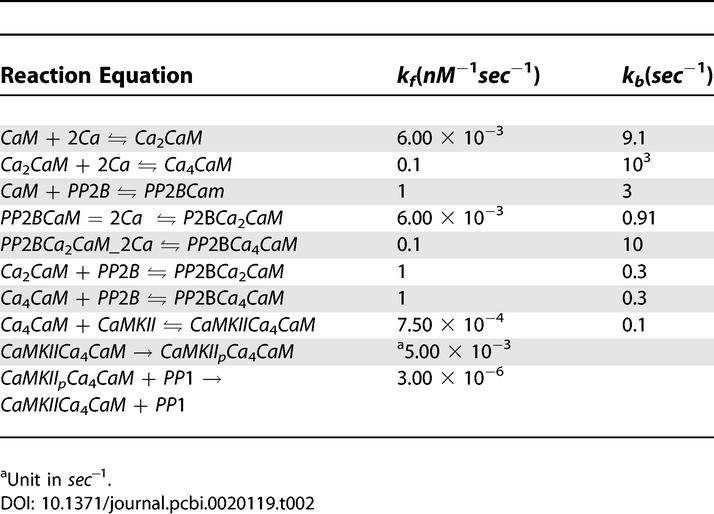
Reactions and Rate Constants of Ca to CaMKII and PP2B Pathway

Several simulations were designed to replicate biochemical experiments in order to compare the model results with “steady-state” experiments, in which the phosphorylation levels were measured after a few minutes. Some simulations were performed for the final adjustment of parameters, and others were performed for model verification. The simulations included 1) a prolonged increase in dopamine concentration, replicating bath application [18,21,22]; 2) a prolonged increase in intracellular calcium concentration, replicating bath application of NMDA and AMPA agonists [16,17]; and 3) a prolonged increase in both dopamine and calcium concentration, replicating the study of Snyder et al. [23].

**Figure 2:**
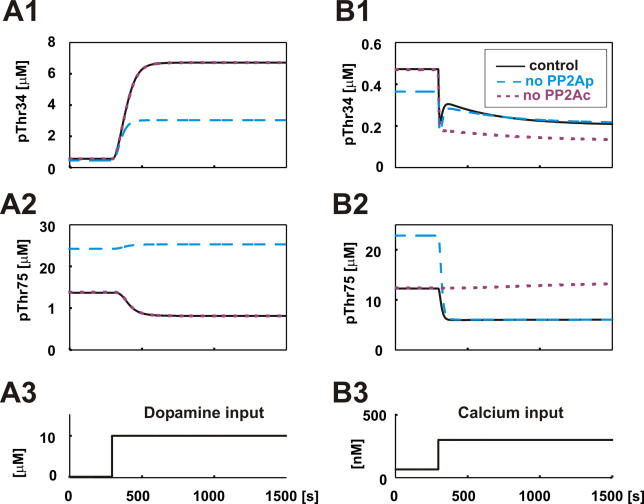
Model Response to Either D_1_ Activation or Calcium Elevation Dopamine elevation at time 300 s (A3) leads to a nine times increase in phosphoThr34 within 2 min (A1, solid line), while phosphoThr75 is reduced to 60% of control (A2, solid line). This is quantitatively in accordance with experimental results. The PKA-dependent activation of PP2A is critical for this behavior; if left out of the model, phosphoThr75 increases instead of decreases (blue dashed line). Calcium-dependent activation of PP2A is not critical to simulate the correct response to D_1_ activation (purple dotted line). Calcium elevation at time 300 s (B3) leads to a reduction in both Thr34 (B1, solid line) and Thr75 phosphorylation (B2, solid line). In contrast to the result in A1 and A2, this response requires calcium-dependent activation of PP2A; if left out of the model (purple dotted line), calcium causes an increase in Thr75 phosphorylation.

A simulated increase in dopamine from 10 nM (the basal level [24]) to 10 μM increases the levels of phosphoThr34 9-fold within 2 min, comparable with the experimental observation of a 6–9-fold increase within 2 to 4 min [17] (Figure 2A1, solid line). In parallel, simulated phosphoThr75 decreases to 60% of basal (Figure 2A2, solid line), in accordance with published data [15]. The mechanism behind the downregulation of phosphoThr75 by dopamine is not known; however, evidence suggests that dopamine acts by increasing the dephosphorylation of phosphoThr75 by PP2A [17]. Other experiments show that PP2A activity is enhanced when it is phosphorylated by PKA [25]. Additional model simulations show that enhanced activation of PP2A via phosphorylation by PKA is required to produce the decrease in phosphoThr75. If this pathway is omitted, a sustained stimulation of the D_1_ receptor instead leads to a small increase in phosphoThr75 (Figure 2A2, dashed line) in contrast to experimental data, though the increase in phosphoThr34 qualitatively remains unchanged. Furthermore, a total removal of PKAc (e.g., by setting the basal dopamine level to zero) causes a significant elevation of phosphoThr75 in the model (unpublished data). This is in good agreement with experimental data, where dopamine depletion causes an increase in phosphoThr75 [26].

A sustained increase in intracellular calcium concentration, as results from prolonged activation of NMDA or voltage-dependent calcium channels, leads to a decrease in the levels of phosphoThr34. In response to a 300-nM calcium signal, both phosphoThr34 and phosphoThr75 decrease to approximately half the basal level within 1 min (Figure 2B, solid lines), in agreement with experimental data [16,17,27]. The decrease in phospoThr34 is due to the calcium-dependent activation of PP2B. In contrast, the dephosphorylation of Thr75 is dependent on calcium activation of PP2A. If calcium activation of PP2A is eliminated, a calcium elevation does not affect the levels of phosphoThr75 much (Figure 2B2, dotted line). It is important to note that this modulation of PP2A by calcium has a minimal effect on the level of phosphoThr34 (Figure 2A1 and 2B1, dotted lines).

**Figure 3:**
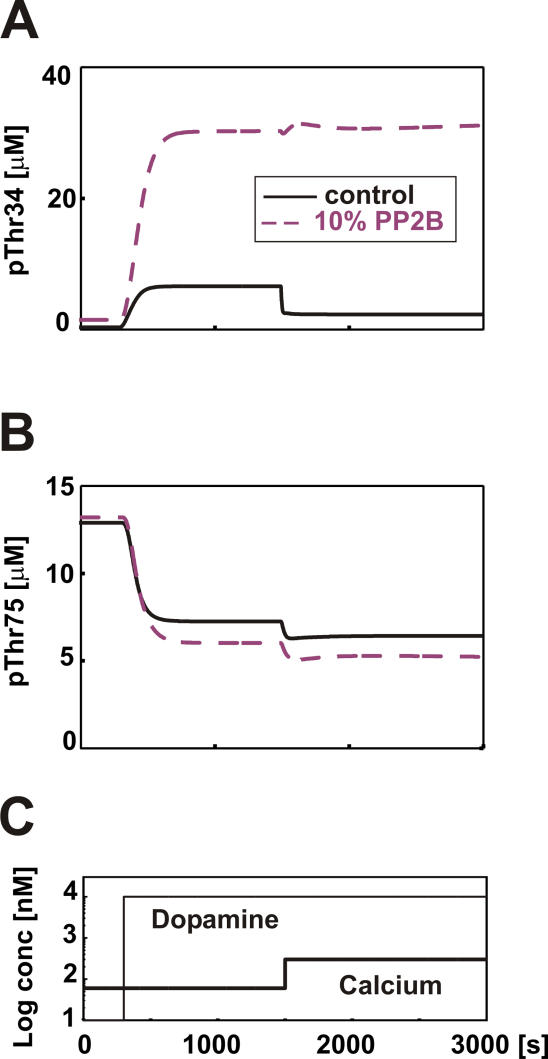
Simulations of Prolonged D_1_ Receptor Stimulation Paired with Calcium Elevation (A) Dopamine-dependent increase in phosphoThr34 starting at time 300 s is reduced when, in addition, calcium is elevated at time 1500 s (solid line). (B) Dopamine-dependent decrease in phosphoThr75 is minimally changed by a subsequent calcium elevation. Reduction of PP2B activity increases the levels of phosphoThr34 and abolishes the calcium-dependent decrease in phosphoThr34 (A, purple dashed line). Furthermore, inhibition of PP2B activity indirectly decreases the levels of phosphoThr75 (B, purple dashed line) because less DARPP-32 is available for phosphorylation on Thr75 since more of it is phosphorylated on Thr34. (C) Time course of dopamine and calcium elevation.

The model is verified by evaluating the change in phosphoThr75 and phosphoThr34 in response to paired dopamine and calcium elevations. As demonstrated experimentally [23], the calcium increase inhibits the increase in phosphoThr34 caused by dopamine (Figure 3A, solid line), and enhances the decrease in phosphoThr75 (Figure 3B, solid line). The decrease in phosphoThr34 caused by elevated calcium is due to PP2B both in the model (Figure 3, dashed lines) and experimentally. If the level of PP2B is decreased to 10% of control (simulating the effect of cyclosporin A for 20 min [27]), the effect of calcium on Thr34 phosphorylation is eliminated.

The steady-state experiments of either dopamine stimulation or calcium stimulation provided values for unconstrained rate constants; however, no rate constants in the model were adjusted while simulating the experiments with paired dopamine and calcium inputs. Thus, comparison of these simulations with experiments represents an authentic verification of the model. Taken together, these results confirm that our model, based on available biochemical data from many independent studies of signalling pathways in the striatum, reproduces the changes in the phosphorylation levels of DARPP-32 at Thr34 and Thr75 produced by activation of dopamine-regulated second messenger pathways and changes in intracellular calcium concentration.

### Response to Transient Inputs Differs from Response to Steady-State Inputs

In vivo, physiological elevations in calcium and dopamine are transient. Dopamine is transiently elevated subsequent to burst firing of nigral or ventral tegmental neurons in response to reward or expectation of reward [3]. Calcium is transiently elevated during striatal up-states [28–30], which are caused by periods of high frequency cortical glutamatergic inputs [31,32]. Since the changes in concentration of most signalling molecules are impossible to measure with this high time-resolution, computer simulations may help elucidate the function of complex second messenger pathways. Thus, the verified model is used to investigate the change in phosphoThr34 and phosphoThr75 in response to transient inputs.

**Figure 4:**
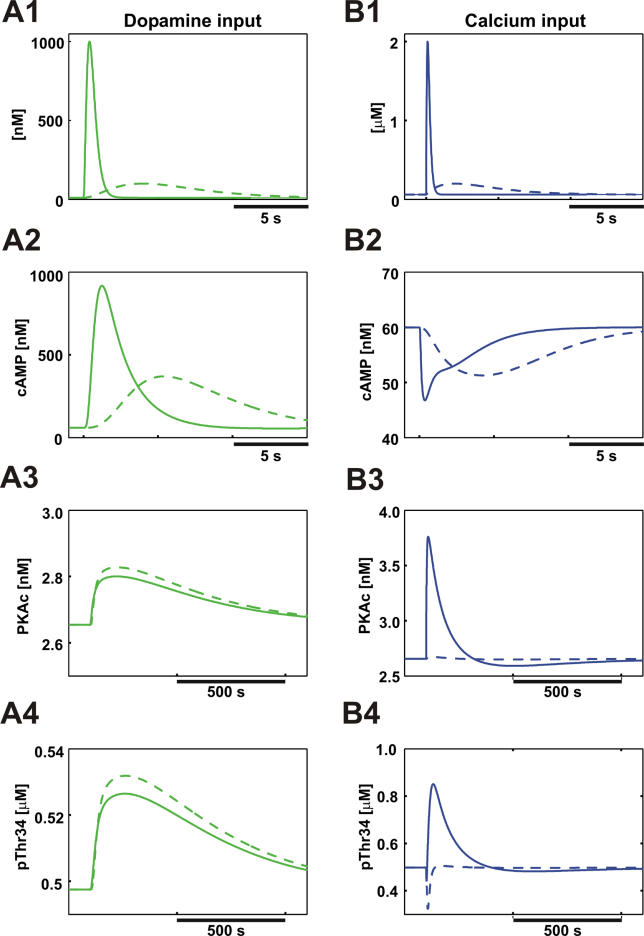
Responses to Transient Dopamine and Calcium Elevations (A1) A brief, high-amplitude (solid line), or slow, low-amplitude (dashed line) dopamine elevation, with approximately equal area under the curve, is used as input to the model network. Dopamine produces an elevation in cAMP (A2), an elevation in PKAc (A3), and an increase in phosphoThr34 (A4). While the cAMP signal follows the dynamics of the dopamine signal, PKAc formation and Thr34 phosphorylation are proportional to total amount of dopamine, as if integrating the cAMP signal because these reactions occur on a slower time scale. (B1) A fast (solid line) or slow (dashed line) transient calcium signal is used as input to the model. Calcium produces a decrease in cAMP (B2) due to both calcium-dependent activation of PDE1 as well as calcium-dependent inhibition of AC5. PDE1 has a larger effect on the maximal cAMP decrease, whereas AC5 has a larger effect on the later part of the cAMP reduction. Despite a decrease in cAMP, the free PKAc concentration (B3, solid line) is transiently increased following a fast and high calcium input, due to the calcium-dependent activation of PP2A, and causes an increase in phosphoThr34 (B4, solid line). A slower calcium input has minor effects on PKAc (B3, dashed line), but causes an initial decrease in phosphoThr34 (B4, dashed line), mostly due to a prolonged activation of PP2B.

A brief dopamine pulse (Figure 4A, solid line; peak = 1 μM, half-width = 0.6 s), comparable to release evoked by a dopamine neuron burst [24,33,34] leads to a small, but sustained increase in free catalytic PKA (PKAc) (Figure 4A3) and a slightly longer increase in phosphoThr34 (Figure 4A4). If the same amount of dopamine is presented as a slow, low concentration signal (peak = 100 nM, half-width = 7 s; Figure 4A1, dashed line), the total amount of cAMP production is similar to that for the brief, high concentration input, although with a different time course that matches the dopamine signal (Figure 4A2). The changes in PKAc level (Figure 4A3) and in phosphoThr34 (Figure 4A4) are almost identical in the brief, high concentration dopamine input: not only are the amounts of free catalytic PKA and phosphoThr34 quite similar, but also the time courses are the same. Thus, PKAc activation acts as a temporal integrator of the cAMP signal.

A transient calcium elevation produces changes in several downstream enzymes (Figure 4B). PP2B is activated by Ca_4_CaM and dephosphorylates DARPP-32 at Thr34. This is the dominant effect of a slow, low concentration calcium signal (peak = 0.2 μM, half with 5 s; Figure 4B1, dashed line), leading to the expected and previously described decrease in Thr34 phoshorylation (Figure 4B4, dashed line). In addition, Ca_4_CaM also activates PDE1, and inhibits adenylate cyclase; both of these factors contribute to reduction of cAMP below basal levels (Figure 4B2). Though the decrease in cAMP concentration is expected to decrease the level of PKAc, this is not observed in the simulations because the calcium-dependent activation of PP2A and consequent dephosphorylation of phosphoThr75 releases PKAc from inhibition in a positive feedback loop involving PKA–PP2A–phosphoThr75 (Figure 1; further explained below). In the case of a fast and high-amplitude calcium signal (peak = 2 μM, half-width = 250 ms; Figure 4B1), the free PKAc even increases transiently despite the decreased cAMP (Figure 4B3, solid line) because the positive feedback loop dominates over the cAMP decrease. The relative importance of this feedback loop is due to the large basal level of phosphoThr75 and thus significant PKAc inhibition. The resulting increase in free PKAc following its release from phosphoThr75 further contributes to an increase in phosphoThr34, with a higher peak value (though shorter half-life) than that caused by dopamine stimulation (Figure 4B4, solid line). These results show that a very brief calcium influx can actually lead to an increase in phosphorylation at Thr34, contrary to the results observed with steady-state calcium signals. This unexpected difference between transient and steady-state effects of calcium is further investigated below with paired transient dopamine and calcium stimuli.

### Repeated Paired Stimuli Produce Enhanced PKAc and phosphoThr34

**Figure 5:**
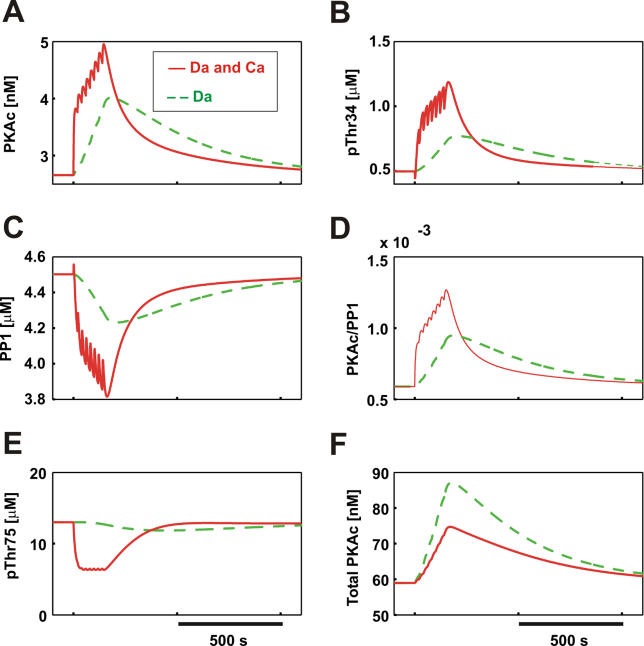
Effect of Simultaneous, Transient Calcium and Dopamine Inputs In all panels, the response to dopamine alone is shown with green dashed lines; the response to paired dopamine and calcium is shown with red solid lines. (A) The cumulative increase in free PKAc following eight high-amplitude, brief dopamine pulses with 20-s intervals, is enhanced when a high-amplitude, brief calcium input is paired with each dopamine pulse. (B) Phosphorylation of Thr34 is enhanced when calcium and dopamine inputs are paired. (C) PP1 inhibition is more pronounced when calcium and dopamine inputs are paired due to the increase in phosphoThr34. (D) The ratio of PKAc to PP1 is enhanced when calcium and dopamine inputs are paired. (E) Some dephosphorylation on Thr75 occurs following a dopamine input due to the PKA dependent activation of PP2A. When in addition calcium is transiently elevated, calcium-dependent activation of PP2A significantly decreases phosphoThr75. (F) The total formation of activated PKA (both free and bound to phosphoThr75) is larger with dopamine inputs alone. This is partly due to the calcium-dependent decrease in the cAMP formation, but also the PKAc interaction with phosphoThr75 is important.

During reinforcement learning, striatal neurons receive paired glutamate and dopamine input. Since several-to-many trials are required for the animal to learn the association, the response of PKAc and phosphoThr34 to repeated paired presentations is of interest. In all the following simulations we have used the same input pulses as described above. Repeated transient dopamine pulses, with temporal properties consistent with reinforcement learning trials (e.g., eight brief pulses at 20-s intervals) produces an accumulation of PKAc (Figure 5A, green dashed line), and significantly increases the phosphorylation of DARPP-32 at Thr34 (Figure 5B). When a fast calcium pulse (as illustrated in Figure 4B1) is given with each dopamine elevation, as would happen with simultaneous release from the glutamatergic and dopaminergic terminals, the activation of PKA is greatly enhanced compared with the response to dopamine alone. The increase in free PKAc due to paired stimuli is 40% greater than the increase observed with dopamine alone, whereas the peak increase in phosphoThr34 is more than doubled (Figure 5A and 5B, solid red lines). The increase in Thr34 phosphorylation in its turn decreases PP1 (Figure 5C), and thus the ratio of PKAc to PP1 is greatly enhanced (Figure 5D) compared with dopamine alone. Since the phosphorylation state depends on the balance between kinases and phosphatases, the increase in PKAc to PP1 is likely to produce a significant change in several target molecules, as has been demonstrated for AMPA receptors [35].

This calcium enhancement of the response to dopamine is in contrast to the effect seen with steady-state inputs in which steady-state calcium inhibits the steady-state dopamine response (compare Figure 3). The calcium pulse given simultaneously with the dopamine input also significantly enhances the decrease in phosphoThr75 (Figure 5E) due to the additional calcium-dependent activation of PP2A, which further elevates PP2A activity. This reduction in phosphoThr75, due to transient dopamine alone, is much smaller than the reduction observed with the high-amplitude (10 μM) steady-state dopamine input used in some experiments (compare Figure 2A2).

In reinforcement learning paradigms, the reward is given after the visual and motor stimuli; thus the dopamine stimulus occurs after the glutamate stimulus. If the reward occurs prior to the visual and motor stimuli, the animal does not learn. Thus, if the second messenger pathways in the model completely explain synaptic plasticity underlying reward learning, the model should be sensitive to interstimulus interval (ISI). Simulations were performed with the dopamine signal occurring prior to calcium (negative ISI) and after the calcium pulse (positive ISI). Large negative ISIs produce an elevation in PKAc comparable with a positive ISI (unpublished data), which is not consistent with behavior. Thus, the model's sensitivity to ISI does not match that of the behavior. This leads to the prediction that although the model reproduces the cooperativity between glutamate and dopamine, additional mechanisms are required to provide temporal sensitivity.

Despite the larger increase in free PKAc with combined calcium and dopamine inputs, the *total* amount of PKAc produced (both free and bound to phosphoThr75) is still larger with dopamine alone (Figure 5F). That less PKAc becomes activated in the presence of increased levels of calcium may be expected because of the calcium-dependent AC5 inhibition as well as calcium-dependent PDE1 activation. Free PKAc levels increase following combined brief calcium and dopamine inputs because less PKAc becomes bound to phosphoThr75. This is further explained by the presence of the PKA–PP2A–phosphoThr75 loop.

### Feedback Loop Modulates Dopamine-Stimulated PKAc and phosphoThr34

**Figure 6:**
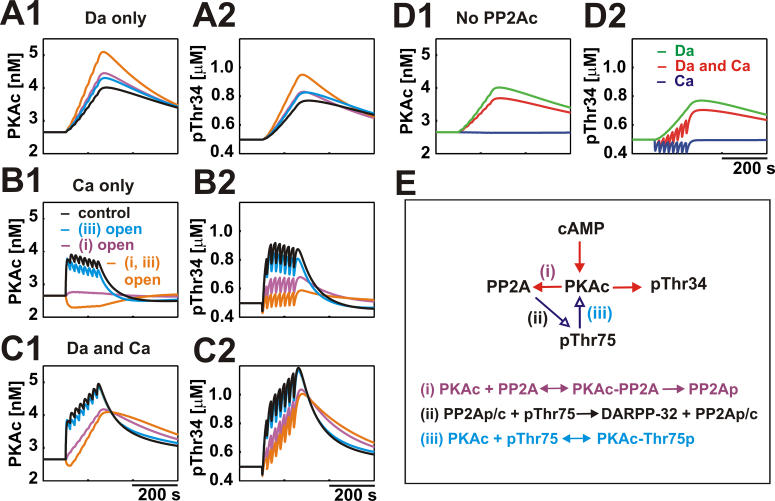
Role of the PKA–PP2A–phosphoThr75 Feedback Loop on PKAc and phosphoThr34 (A) Eight high-amplitude, brief, dopamine pulses, 20 s apart, lead to a larger buildup of free PKAc (A1) or Thr34 (A2) compared with control (black) if the reaction rates are set to zero in the PKA phosphorylation of PP2A only (purple; reaction (i) in (E) opened), in the phosphoThr75 inhibition of PKA only (light blue; reaction (iii) in (E) opened), or in both reactions (orange). Thus, both reactions (i) and (iii) in the PKA–PP2A–phosphoThr75 loop work as a sink for the PKAc signal. (B) High-amplitude, fast calcium inputs lead to a decreased level of free PKAc (B1) and Thr34 (B2) if reactions (i) and (iii) are prevented. Here the PKA–PP2A–phosphoThr75 loop behaves as a positive feedback loop on the PKAc concentration. (C) Paired fast calcium and dopamine elevations enhance PKAc (C1) and phosphoThr34 levels (C2) when the PKA–PP2A–phosphoThr75 loop is present (black lines), but decrease PKAc and phosphoThr34 when reactions (i) and (iii) are eliminated (orange lines). (D) Activation of PP2Ac by binding to calcium is required for the stimulatory effect of calcium on PKAc and phosphoThr34. Binding of calcium to PP2A shifts equilibrium of reaction (i) to the substrates, thus PKAc dissociates from PP2A, and shifts the equilibrium of reaction (iii) to the substrates, thus PKAc dissociates from phosphoThr75. If calcium activation of PP2A is prevented, dopamine alone (green lines) results in more PKAc (D1) and phosphoThr34 (D2) than paired calcium and dopamine (red lines). Also, calcium alone (blue lines) produces no significant response in PKAc and even causes a decrease in phosphoThr34.

PKAc enhancement of PP2A activity (reaction (i) in Figure 6E); PP2A dephosphorylation of phosphoThr75 (reaction (ii)); and subsequent decreased inhibition of PKAc (reaction (iii)) can act on PKAc as a positive feedback loop. This loop is believed to enhance the levels of free PKAc, which would then enhance phosphorylation of DARPP-32 on Thr34 in response to dopamine stimulation [13]. To test this hypothesis, model simulations, using the same dopamine and calcium pulses as described above, are repeated with this positive feedback loop opened, by setting to zero the rate constants for reactions (i) and (iii). Removing the phosphorylation of PP2A by PKAc should decrease the modulation of phosphoThr75, and reduce the disinhibition of PKAc.

Surprisingly, opening the feedback loop increases the concentration of both PKAc (Figure 6A1, orange line) and phosphoThr34 (Figure 6A2, orange line) compared with the control condition (Figure 6A, black lines). Eliminating just the phosphorylation of PP2A by PKAc (reaction (i), magenta lines), or eliminating just the inhibition of PKA by phosphoThr75 (reaction (iii), light blue lines), produces a small increase in PKAc, but eliminating both reactions produces a dramatically larger increase in PKAc. The supralinear effect of these two feedback loop reactions suggest that the apparent inhibitory effect of the loop following a transient dopamine input is due to a change in equilibrium in the reactions involving PKAc. As more PKAc is formed, much of it is bound to PP2A in the enzyme-substrate complex, as well as to phosphoThr75 in the inhibitory complex (Figure S1A). Thus these two forms of bound PKA act as a sink for free PKAc, dampening the stimulatory effect of dopamine on the increase in the free PKAc levels.

Eight transient calcium pulses cause an increase in both PKAc and phosphoThr34 (Figure 6B, black lines) that is comparable to the increase caused with eight pulses of dopamine alone (compare Figure 6A). Because calcium increases PP2A activity, it is possible that the feedback loop is involved in this response. Simulations show that when the feedback loop is partly or completely opened (Figure 6B), the response of PKAc and phosphoThr34 to a brief calcium input is dramatically reduced. Opening up both reactions in the loop actually transforms the effect of calcium on PKAc into inhibition (due to inhibition of AC5 and activation of PDE1). Thus, binding of calcium to PP2A has two important effects. First, it reduces the amount of unbound PP2A, and thus reduces the substrate available for PKAc binding. This decrease in PKAc–PP2A (Figure S1B) shifts the equilibrium of reaction (i), thus PKAc dissociates from PP2A. Second, the enhanced PP2Ac activity dephosphorylates phosphoThr75 and disinhibits PKAc. The quantity of PKAc–phosphoThr75 decreases (Figure S1B), reflecting a shift in the equilibrium of reaction (iii), as PKAc dissociates from phosphoThr75. The stimulatory effect of calcium on PKAc requires the activation of PP2Ac, dephosphorylation of Thr75, and disinhibition of PKAc. Opening reaction (i) eliminates the reduction of PKAc-PP2A by calcium, and opening reaction (iii) eliminates the reduction of PKAc-phosphoThr75 by calcium.

When brief dopamine and calcium pulses are given together (eight paired pulses at 20-s intervals), the calcium-dependent enhancement through the PKA–PP2A–phosphoThr75 loop dominates (Figure 6C). The combination of dopamine and calcium produces a smaller quantity of the PKAc–PP2A complex than dopamine alone (Figure S1C). When reaction (i) and (iii) are set to zero, the reduction in PKAc–PP2A and PKAc–phosphoThr75 complexes are smaller than in the control case. Thus, when rates of reaction (i) and (iii) are set to zero (opening the loop), both PKAc and phosphoThr34 increase less compared with when the loop is fully active.

In the Thr to Ala mutant mouse [36], in which Thr75 cannot be phosphorylated, increasing dopamine levels no longer affect downstream processes such as CREB phosphorylation. To replicate this experiment, phosphoThr75 is set to zero in the model. The new equilibrium has a different basal level of PKAc and phosphoThr34 (unpublished data); however, the increase in PKAc due to paired stimuli is similar to the case when reaction (iii) is eliminated, consistent with experimental results [36,37]. In summary, the PKA–PP2A–phosphoThr75 feedback loop is important for increasing levels of free PKAc and phosphoThr34, but only when transient calcium elevations are present, and not in response to transient inputs of dopamine alone.

Calcium activation of the feedback loop is essential for the enhancement of PKAc and phosphoThr34 in response to paired stimuli. This implies that if calcium-dependent activation of PP2A is eliminated, but the PKA–PP2A–phosphoThr75 loop is kept intact, pairing calcium and dopamine will give smaller elevations of free PKAc and phosphoThr34, than dopamine alone. Furthermore, calcium stimulation alone will reduce both PKAc and phosphoThr34. This is partly due to calcium-dependent inhibition of AC5 and calcium-dependent activation of PDE1, but also because now the PKA–PP2A–phosphoThr75 loop instead works as a sink for PKAc. This prediction is tested with simulations in which the calcium-dependent enhancement of PP2A is eliminated (rate constants set to zero). Figure 6D shows that now calcium alone (blue lines) produces a decrease in both PKAc and phosphoThr34 (qualitatively similar to that observed with the feedback loop eliminated), and calcium paired with dopamine inhibits the production of PKAc and phosphoThr34 (red lines), again similar to that observed with the feedback loop eliminated. Thus, without the calcium-dependent PP2A augmentation, a transient dopamine input alone is predicted to increase both PKAc activation and phosphoThr34 more effectively than dopamine paired with calcium.

### Role of Calcium Dynamics

**Figure 7:**
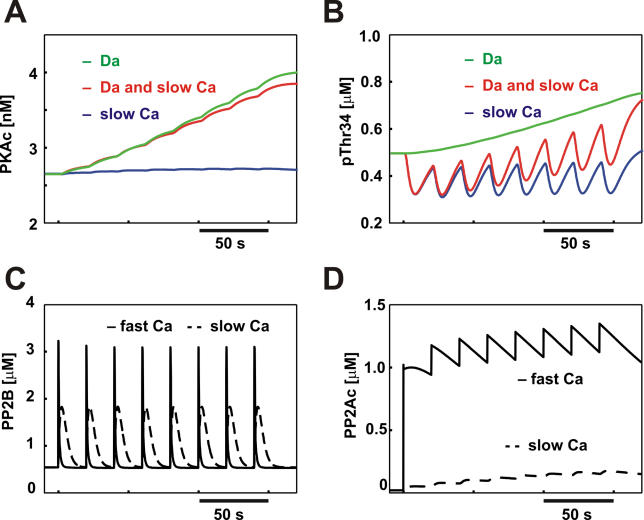
Consequences of Pairing Slower Calcium Signals with Dopamine Inputs (A) The cumulative increase of free PKAc following eight high-amplitude, brief dopamine inputs (green line) is a bit larger than if a slow, low-amplitude calcium signal is paired with the brief dopamine inputs (red line). Also the slower, low-amplitude calcium input alone (blue line) does not give any significant increase in PKAc. (B) Pairing slow, low-amplitude calcium inputs with dopamine inputs (red line) reduces the cumulative increase in phosphoThr34 compared with a dopamine input alone (green line). A decrease of phosphoThr34 is seen following a calcium input alone (blue line). (C) The decrease in phosphoThr34 due to the slow, low-amplitude calcium input is due to a prolonged activation of PP2B (dashed line) compared with when a brief, high-amplitude calcium input is given (solid line). (D) Furthermore, the calcium-activated form of PP2A (PP2Ac) is significantly less activated following a small but slower calcium input (dashed line) than if a high-amplitude calcium input is given (solid line).

Many different mechanisms control calcium dynamics in spiny projections neurons. Brief calcium elevations are produced by synaptic activation and backward propagating action potentials. A slower and lower calcium elevation might result from calcium release from intracellular stores. This is one proposed mechanism by which the postsynaptic dopamine D_2_ receptor acts [38]. Therefore, to evaluate the effect of calcium dynamics, simulations were repeated using a slower and lower calcium elevation (as in Figure 4B1, dashed line). When slow calcium inputs are paired with an increase in cAMP, the buildup of free PKAc is unchanged or minimally decreased compared with cAMP increase alone (Figure 7A), instead of enhanced as when faster, larger calcium pulses are used (compare Figure 5). In addition, the buildup of phosphoThr34 is decreased (Figure 7B).

These differences between a brief, high-amplitude calcium input and a slower, low-amplitude calcium input are due to the different effects that these calcium signals have on the level of PP2B and PP2A activation. Both the slow and the fast calcium inputs activate PP2B, though with a more prolonged time course if a longer calcium elevation occurs (Figure 7C, dashed line). But only the briefer and high concentration calcium activates PP2A significantly (Figure 7D, solid line). The slow calcium pulse therefore does not enhance dephosphorylation at Thr75, and thus does not enhance PKAc.

### The Main Simulation Results Are Independent of Model Parameters

**Figure 8:**
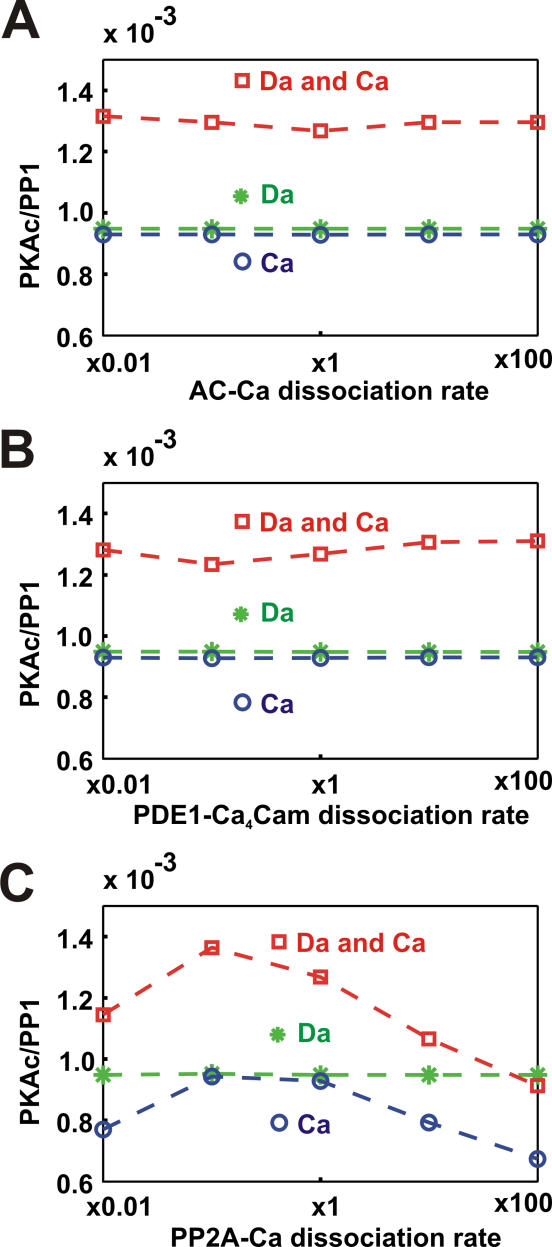
Effects of Dissociation Rate on Calcium Enhancement of Dopamine-Induced PKAc Elevation and PP1 Suppression In all panels, the response is the maximal ratio of PKAc to PP1. All panels show the effect of dissociation rate on the response to dopamine alone (green dashed line, stars), calcium alone (blue dashed line, circles), and paired dopamine and calcium (red dashed line, squares). (A) Response is independent of rate of calcium-dependent AC5 inhibition. (B) Response is independent of rate of Ca_4_CaM binding to PDE1. (C) Response is slightly sensitive to the rate of calcium binding to PP2A: for very fast (*100) calcium dissociation rate, the enhancing role of calcium decreases because the activation of PP2A is too brief to dephosphorylate significant amounts of phosphoThr75 following brief calcium inputs.

The main findings from these simulations suggest that the presence of the PKA–PP2A–phosphoThr75 loop causes increased formation of free PKAc as well as phosphoThr34 if a dopamine input is paired with a transient calcium elevation. To investigate the robustness of this result, simulations are repeated with variations in the least constrained parameters. Though most rate constants are constrained by direct measurements of affinity, the dissociation constants of some reactions have not been reported. In particular, the dissociation rate for calcium binding to AC5, calcium–calmodulin binding to PDE1, and calcium-dependent activation of PP2A is not known. Thus, simulations are repeated using a range of dissociation rate constants for each of these reactions. Figure 8A and 8B shows that, over a large range of dissociation rates for calcium binding to AC5 or Ca_4_CaM binding to PDE1, the PKAc elevation and PP1 suppression in response to a dopamine signal is enhanced when dopamine is paired with calcium. The model is mildly sensitive to the rate of calcium-dependent enhancement of PP2A activity. If the activation and deactivation kinetics are much faster (100× as fast as control values), paired dopamine and calcium inputs are no more effective than dopamine alone (Figure 8C). With a fast dissociation rate, the deactivation of the calcium-activated PP2A (PP2Ac) occurs too rapidly to allow PP2Ac to dephosphorylate phosphoThr75 during brief calcium elevations. Also, if the calcium activation is very slow, then very little calcium-dependent activation of PP2A occurs during the brief duration of the calcium inputs, and the effect of paired stimulation decreases. A 100× slower kinetics of calcium-induced PP2A stimulation is, however, not in accordance with results from Nishi et al. [17], in which a significant dephosphorylation of phosphoThr75 occurs within 5 min.

**Figure 9:**
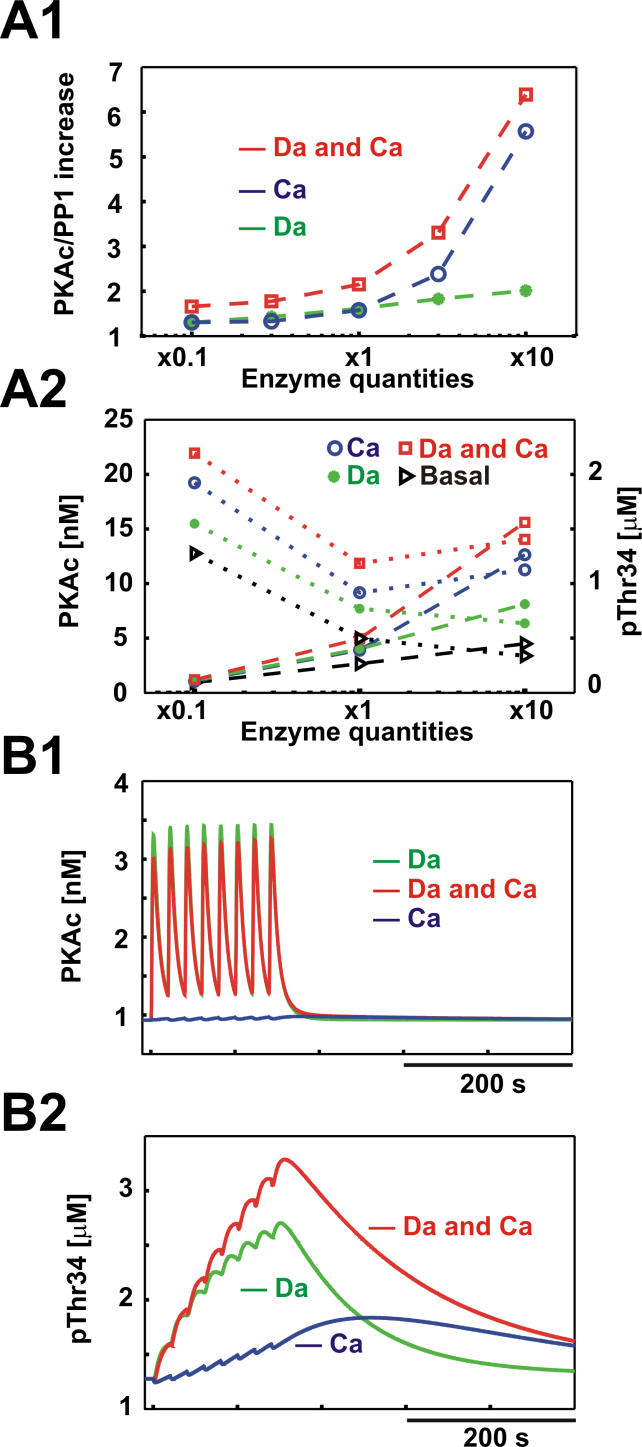
Role of Altered Enzyme Quantities on the Integration Properties of DARPP-32 (A) Total concentrations of PKA, PP2B, PP2A, and cdk5 all are varied together between 0.1–10× the control level. (A1) Independent of this variation, pairing of calcium and dopamine (red, squares) is more effective than either input alone (green dots or blue circles). (A2) The basal levels of PKAc (black dashed line, triangles) as well as phosphoThr34 (black dotted line, triangles) vary with enzyme quantity. A decrease in enzyme quantity produces a decrease in the change of PKAc during stimulation (dashed line) and an increase in the phosphoThr34 levels (dotted line). Nonetheless, in all cases a simultaneous calcium and dopamine input is most effective in elevating both PKAc and phosphoThr34. (B) The role of DARPP-32 in signal integration is independent of PKAc. Enzyme quantities were lowered to 10% of control and all the reaction rates in the PKA reactions were increased 100× to make the PKAc formation follow the cAMP signal more effectively. (B1) The PKAc increase is slightly larger with dopamine alone and decreases almost to basal level in between transient dopamine pulses. (B2) Thr34 phosphorylation increases with successive paired stimuli, and is higher for paired calcium and dopamine inputs.

Though the quantity of DARPP-32 has been estimated [37], enzyme quantities are typically measured from tissue volumes which include both neurons and non-neuronal cells. The actions of anchoring proteins for molecules such as PKA suggest that the effective quantities of these enzymes in the synapse may be much higher than published estimates [39]. Thus, simulations are repeated using both higher and lower quantities of the enzymes (PKA, cdk5, PP2A, PP2B) to determine to what extent the results are sensitive to these parameters. Figure 9A1 shows that, even with a 10-fold increase or decrease in enzyme quantity, calcium paired with dopamine produces a larger PKAc-to-PP1 ratio than dopamine alone. Simulations in which the quantity of single enzymes or pairs of enzymes was changed did not appreciably change the results. Since the ratio PKAc:PP1 controls AMPA channel phosphorylation, this larger increase in PKAc:PP1 would translate into more LTP due to paired stimulation.

Nonetheless, the signalling network exhibits changes in activity due to the change in enzyme quantity. Specifically, both the basal and stimulated levels of PKAc are altered. Figure 9A2 (dashed lines) shows that both basal and stimulated PKAc decrease when enzyme quantities are decreased to 10% of the control values, and that both basal and stimulated PKAc increase when enzyme quantities are increased. In contrast, the basal and stimulated levels of phosphoThr34 increase with a decrease in enzyme concentration (Figure 9A2, dotted lines). In summary, though the basal level of PKAc:PP1 changes with a change in enzyme quantity, the basic principle, that paired stimulation produces a larger increase than either calcium or dopamine alone, is robust to enzyme quantity.

To further demonstrate the importance of DARPP-32 in the integration of signalling pathways, simulations were repeated with parameter variations designed to make DARPP-32 independent of its regulatory molecules. The total concentration of PKA, PP2A, PP2B, and cdk5 were set to 10% of control values, and the dynamics of the PKA system were accelerated, i.e., the rate constants for all reactions involving PKA were increased 100-fold. These changes decreased the signal integration capability of PKA, and exposed the signal integration capability of DARPP-32.

As seen in Figure 9B1, the amplitude of the PKAc response increases since it is activated more efficiently by cAMP. In addition, PKAc decreases almost to basal level between each pair of dopamine pulses. In contrast, phosphoThr34 accumulates during the eight pulse stimulus (Figure 9B2), just as in the control model. More importantly, though calcium paired with dopamine produces a slight decrease in free PKAc, it produces an increase in phosphoThr34 (Figure 9B2). Thus, independent of the ability of PKAc to integrate dopamine and calcium signals, phosphoThr34 also integrates dopamine and calcium signals.

Last, we evaluated whether the results were contingent upon the high DARPP-32 concentration. Reducing DARPP-32 to 10 μM or 1 μM produced a change in the basal level of PKAc, phosphoThr34, and PP1, but produced very little change in the ratio PKAc:PP1. More importantly, the increase in PKAc:PP1 in response to paired stimulation remains greater than the response to dopamine alone (Figure S2A), similar to simulations in which other enzymes were increased. The relative increase due to paired stimulation compared with the sum of dopamine and calcium inputs alone is larger with the higher DARPP-32 concentration compared with the lower, suggesting that the high concentration of DARPP-32 in striatal neurons that has been measured may have a physiological relevance. Nonetheless, these simulations further confirm the robustness to parameter variations of the main result, namely that combined transient inputs are more effective.

## Discussion

To better understand the complex intracellular signalling networks underlying synaptic plasticity and reinforcement learning, we have developed a model of the calcium and cAMP signalling cascades that regulate DARPP-32 phosphorylation. The model is based on published biochemical data: the simulated regulation of DARPP-32 phosphorylation by sustained G-protein receptor activation and calcium influx fits experimental data, increasing and decreasing phosphoThr34, respectively (Figures 2 and 3).

Interestingly, our model suggests that the effect of transient calcium influx is very different from sustained calcium elevation and that short bursts of calcium influx actually increase DARPP-32 phosphorylation on Thr34 (Figure 4). More significantly, combined dopamine and glutamate stimulation produces a larger activation of PKA and inhibition of PP1 than dopamine alone (Figure 5). This is potentially important as both nigrostriatal dopaminergic input and corticostriatal glutamatergic input often terminate on heads and necks of dendritic spines [40,41]. Since phosphorylation state depends on the balance between kinases and phosphatases, the increase in PKAc to PP1 is likely to produce a significant change in several target molecules, such as GABA [42] receptors, as well as sodium [43] and calcium channels [44], which will modify the subsequent response to synaptic stimulation. In particular, D1 receptor activation, producing PKAc, phosphorylation of DARPP-32, and subsequent inhibition of PP1 activates kinases that phosphorylate AMPA channels [45], leading to synaptic potentiation. Simulations further demonstrated that the dephosphorylation of DARPP-32 at Thr75 can enhance PKA activity through a disinhibition loop, as has been suggested, but only during calcium influx alone or in combination with dopamine. This loop instead works as a sink when transient dopamine inputs are used and dampens the increase in free PKAc (Figure 6).

### The Role of DARPP-32 in Reinforcement Learning and LTP

The concomitant increases in calcium and dopamine are behaviourally relevant stimuli in that they occur during reinforcement learning. Visual stimuli and motor action cause corticostriatal fibers to release glutamate; the subsequent reward is accompanied by dopamine release from nigrostriatal fibers. Thus, it is possible that during reinforcement learning, the calcium elevation produced by glutamate stimulation, together with cAMP produced by dopamine stimulation, induces synaptic plasticity, which underlies learning the association between the visual stimulus and reward.

Theoretical models of reinforcement learning, e.g., temporal difference learning [8,46,47], posit that the striatum learns which among multiple actions produces the greatest future rewards. Memories of rewarded motor actions may be stored as potentiated corticostriatal synapses. During learning, those synapses that release glutamate when dopamine arrives are potentiated. Subsequently, glutamate stimulation alone evokes output from those neurons encoding the reinforced motor action.

Experimental support for temporal difference learning is rather strong. Experiments from striatal slices show that release of glutamate from corticostriatal fibers produces LTP in the presence of dopamine. Neither dopamine alone nor cortical stimulation alone produces LTP [48–50], and corticostriatal LTP is blocked in animals lacking DARPP-32 [49]. Activated PKA is known to mediate phosphorylation of Ser845 on the AMPA receptor GluR1 subunit [51,52], leading to insertion of AMPA receptors on the cell surface [45,53,54].

The model results presented here help explain the mechanisms underlying temporal difference learning. The prevailing view, based on biochemical experiments used to derive our model, has been that glutamate and dopamine have opposite effects on the PKA signalling cascade. Calcium decreases PKA activity, and increases PP1 activity, whereas dopamine does the opposite. In contrast, the model shows that transient calcium stimulation can enhance the PKA signalling cascade. Thus, when transient dopamine and glutamate are given together, the increase in the ratio of PKA to PP1 activity is enhanced compared with when dopamine is given alone (Figure 5). Thus, model simulations suggest that PKA activation, phosphorylation of DARPP-32 at Thr34, and subsequent PP1 inhibition, produced by conjunctive glutamate and D_1_ receptor stimulation, can produce LTP by increased phosphorylation of AMPA receptors. This suggests that the cellular mechanisms leading to temporal difference learning is the enhanced stimulation of PKA with paired calcium and dopamine stimuli. Thus, at a network level, when a subset of spiny projection neurons are activated by glutamate and dopamine simultaneously, corticostriatal synapses of those spiny projection neurons are strengthened; subsequently, glutamate stimulation alone evokes output from the correct neurons.

The dynamics of the calcium transient controls the effect of calcium on the dopamine induced PKAc elevation. A persistent calcium elevation has a minimal effect on PKAc and decreases phosphoThr34, whereas a brief transient calcium elevation produces an increase in both PKAc and phosphoThr34. These results lend significance to the calcium imaging studies [29,30] which show that a backward propagating action potential enhances the calcium elevation during an up-state. This suggests that synaptic plasticity may require not only coincident glutamate and dopamine inputs, but also sufficient spiny projection neuron activity to produce a backward propagating action potential and the accompanying fast, high-amplitude calcium transient. This requirement for glutamate, dopamine, and a backward propagating action potential is the “three-factor rule” [7,55]**.**

Nonetheless, the response to paired calcium and dopamine stimuli did not match the sensitivity to ISI as seen with behavior. Several other mechanisms within spiny projection neurons may be responsible for this sensitivity.

1) G protein coupled receptors such as mGluR and mAChR produce diacylglycerol and inositol triphosphate leading to calcium release from intracellular stores. Calcium release plays a role in sensitivity to ISI in cerebellar Purkinje cells [56,57]; and diacylglycerol activates protein kinase C, which releases calmodulin (CaM) from neurogranin [58]. When included in future versions of the medium spiny neuron model, these mechanisms may produce sensitivity to ISI.

2) In medium spiny neurons, various receptors and enzymes are localized to different compartments. For example, calcium influx through NMDA receptors occurs at the postsynaptic density, and dopamine receptors are distributed not only on the spines, but also on the dendritic shaft [59]. Anchoring of molecules such as CaMKII and PKA at the postsynaptic density [39,60] and diffuse distribution of other molecules such as PP2A implies that diffusion plays a role in the interaction between dopamine and calcium-activated pathways. This diffusion, by introducing temporal delays, may produce sensitivity to ISI. In addition to these intraneuronal mechanisms, it is possible that network interactions or presynaptic dopamine receptors may produce the ISI sensitivity observed behaviourally.

### Transient and Sustained Calcium Have Different Effects on DARPP-32 Phosphorylation at Thr34

The nature of biochemical experiments makes it difficult to measure phosphorylation state with high time-resolution. Thus, the simulation result that transient calcium enhances phosphoThr34 is a prediction of the model (Figures 4–6). Recently, Nishi et al. used glutamate for brief stimulations (15 s) and did see an increase in phosphorylation at Thr34 [61]. Although they suggest this increase is mediated indirectly, via release of nitrous oxide and production of cGMP, model simulations suggest that it can also be an effect of calcium increase through NMDA receptors.

The mechanism of action of D_2_ receptors in the striatum is unclear, since both inhibition of adenylate cyclase [62] and release of intracellular calcium [38,63] has been proposed. We show here that slow calcium transients, as occurs with calcium release from intracellular stores, are able to dephosphorylate Thr34. This may explain observations that activation of D_2_ receptors decreases phosphoThr34 and decreases the phosphorylation of PP1 substrates [21,64].

#### The dual role of Thr75 phosphorylation.

**Figure 10:**
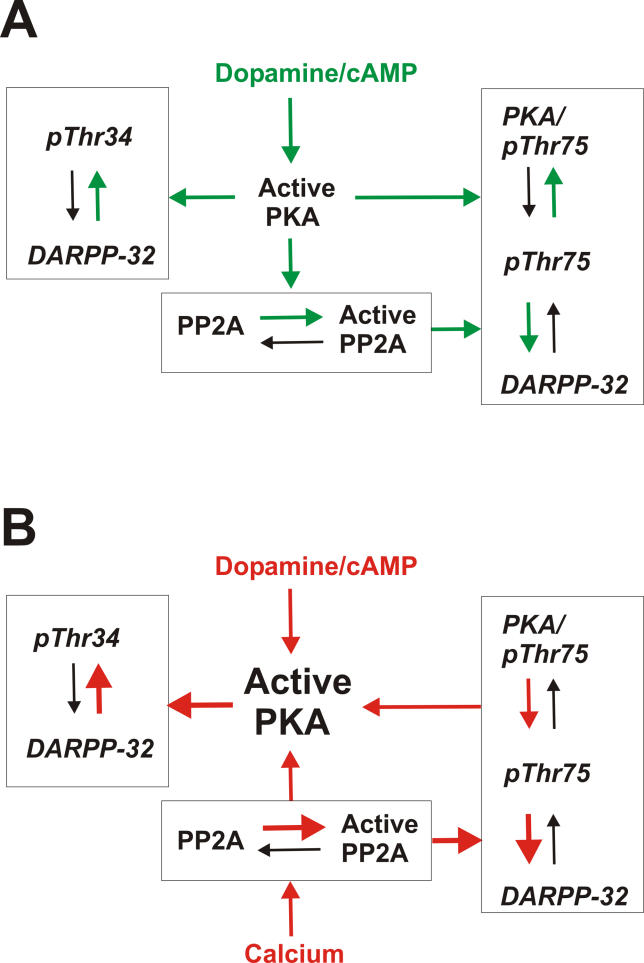
Schematic Drawing of the Reactions Involved in the PKA–PP2A–phosphoThr75 Loop When dopamine stimulates PKA, a substantial amount of the free catalytic subunit gets bound to PP2A and phosphoThr75, thus dampening the stimulatory effect of dopamine on PKA (A). Calcium stimulation and the formation of the calcium-activated PP2A affects this loop in two ways: 1) by increasing dephosphorylation of phosphoThr75, thus reducing inhibition of PKA; and 2) less PP2A is available for PKA binding, thus shifting this equilibrium to increase the amount of active PKA (B).

The disinhibition of PKA when phosphoThr75 is dephosphorylated has been suggested to be an important part of the potentiating effect of DARPP-32 on PKA activity [65]: PKA enhances PP2A activity via phosphorylation; then PP2Ap dephosphorylates phosphoThr75 at an accelerated rate and the decrease in phosphoThr75 leads to more active PKA. Nonetheless, during transient dopamine inputs alone, the loop acts as a sink, in which binding of PKAc to PP2A or phosphoThr75 actually decreases the amount of free PKAc, and thereby decreases the ability of PKAc to phosphorylate DARPP-32 on Thr34. In contrast, the stimulation of PP2A through calcium shifts the balance of the loop, increasing dephosphorylation of phosphoThr75 and thus disinhibiting PKA. Figure 10 illustrates these two alternative modes of operation of this loop. The relative changes in the reaction flows are dependent on the biochemical reactions involving PKAc (Figure S1 further illustrates the quantitative role that PP2A substrate depletion and phosphoThr75 inhibition of free PKAc play in the feedback loop). An important difference between transient and steady-state calcium inputs is predicted by the model, due to the relative activity of PP2A versus PP2B. Steady-state calcium produces a large increase in PP2B, dephosphorylating phosphoThr34; in contrast, large, transient calcium elevations produce an increase in PP2A activation and consequent disinhibition of free PKAc due to phosphoThr75 dephosphorylation. The net effects of these different calcium inputs on the PKAc:PP1 balance are summarized in Figure S2B. If calcium is prevented from binding to and enhancing PP2A activity, then a transient calcium stimulation does not increase PKAc activity, but instead decreases PKAc. Similarly, if PP2Ac is prevented from dephosphorylating DARPP-32 on Thr75, then instead of an increase in PKAc activation following calcium stimulation, the amount of activated PKAc is decreased (compare Figure 6D).

One validation of the importance of phosphoThr75 in the action of calcium can be found in the study of different psychotomimetics in mice in which DARPP-32 has been genetically modified at either Thr34 or Thr75 so that these sites cannot be phosphorylated [36]. Phencyclidine (PCP), a drug that affects glutamate transmission and calcium influx, is the only drug whose effects are modified in the mice where Thr75 could not be phosphorylated, whereas lysergic acid diethylamide (LSD), which alters serotonergic transmission, and amphetamine, which alters dopamine transmission, have the usual effect on behavior in these animals.

### Regulation of Phosphatases Shape the Response of the Signalling Networks

The regulation of kinases such as PKA and CaMKII has been studied in great detail, whereas much less attention has been given to the regulation of phosphatase activity. Nonetheless, our model, together with other modeling work [66–68], clearly suggests that the regulation of protein phosphatases is very important to shape the response in signalling networks. In the DARPP-32 signalling pathway, dynamic regulation of the level of phosphoThr75 by PP2A is essential. To reproduce experimental data on DARPP-32 phosphorylation, the regulation of PP2A by both calcium and dopamine is required. Although only PKA regulation has been directly proven, experiments show that glutamate inhibits phosphoThr75 [17] via a PP2A and calcium-dependent pathway. The importance of this pathway for mediating the cooperative action of calcium and dopamine suggests that future experimental demonstration and kinetic characterization of calcium-activated PP2A is critical.

### Results Are Robust to Variations in Parameters

A common criticism of this type of model is that simulation results are sensitive to parameters, and that insufficient data is available to constrain the parameters. To preempt this criticism, several simulations demonstrate that the present model is robust to variation in the unconstrained parameters.

The result that a transient calcium elevation can increase phosphoThr34 was shown with a previous model [69]. This earlier result was critically dependent on the presence of a Ca_4_CaM-stimulated form of AC; however, the predominant form of AC in striatum is type 5 [70,71], which is not stimulated by calcium. Incorporating the effect of phosphoThr75 and the regulation of PP2A in our model made the stimulatory effect of calcium robust to parameter variations (Figure 8).

A potential source of error in this model is the concentration of various components. Although the concentration of many of the enzymes has been published, the data usually comes from crude homogenates, and does not take into account compartmentalization or scaffolding of molecules. As shown in Figure 9, however, the most important conclusions from this model are qualitatively robust, even when enzyme concentrations are varied 10-fold.

Experiments have not shown directly whether the phosphorylation of Thr34 influences the rate of phosphorylation of Thr75. It is known, however, that phosphorylation of Ser 102 and Ser 137 influences the rate of phosphorylation of Thr34 [72,73], indicating that intramolecular mechanisms are important. Model simulations justified the constraint that DARPP-32 can only be phosphorylated at one threonine residue at a time. An alternative model, in which phosphorylation rate constants at one threonine site are lower (1/5) when the other threonine is phosphorylated gave very similar results (unpublished data). Nonetheless, this is an issue that needs to be addressed experimentally.

In conclusion, our results support the idea that subcellular processing, such as coincidence detection, signal amplification, and decision making, based on the interactions within the intracellular networks, are essential for control of neuronal activity [74]. Some of these critical interactions control whether learning and synaptic plasticity will occur in different brain regions (see, e.g., [57,75]). Since it is exceedingly difficult to visualize the dynamics of intracellular signalling pathways, modeling is an important adjunct method for formulating hypotheses regarding the critical steps in these pathways [69,76]. New imaging techniques such as Fluorescence Resonance Energy Transfer (FRET) [77,78], rather than removing the need for dynamic modeling, instead provide essential constraints that improve the veracity of such models. Future modeling approaches that incorporate both this data and compartmentalization of enzymes due to anchoring proteins [35,79–81] will have an increased ability to formulate hypotheses regarding key molecules controlling neuronal dynamics and plasticity in the basal ganglia.

## Materials and Methods

All reactions in the model are described as protein–protein interactions:

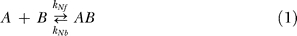
or as enzymatic reactions written in the Michaelis–Menten form which assumes a nonreversible catalytic step:

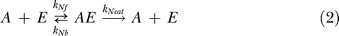



The concentration of the substrate A in reaction 1 is described using a first-order differential equation of the form:

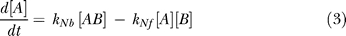



Solution of this equation requires initial concentrations and the forward (k_Nf_) and backward (k_Nb_) rate constants for reaction N. For cascades of reactions, equations are derived by summing terms describing all reaction pathways leading toward or away from a particular molecule. For example, the rate of change of concentration of AE in Equation 2 is given by:

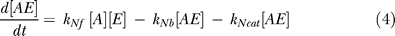
[76,82].

For protein–protein interactions, the equilibrium constant, K_d_, is defined as the ratio of backward-to-forward rate constant: k_Nb_/k_Nf_. For enzymatic reactions, k_Ncat_ defining the last, catalytic step, is the rate at which product appears (sometimes called V_max_), and the affinity, K_M_ = (k_Ncat_ + k_Nb_)/k_Nf_. When k_Nb_ is not known explicitly, k_Nb_ is defined as 4 k_cat_ [76,83]. As explained below, all parameters are derived from experimentally measured constants found in publications, or carefully inferred from indirect biochemical studies.

The reactions and rate constants in the model are summarized in Tables 1–3 and Figure 1. The total concentrations of molecules are summarized in Table 4. The equations were programmed in XPPAUT (http://www.math.pitt.edu/~bard/xpp/xpp.html) and run under the UNIX operating system. Simulations used the numerical integration method called “stiff” with a time step of 0.01–0.1 s.

**Table 3:**
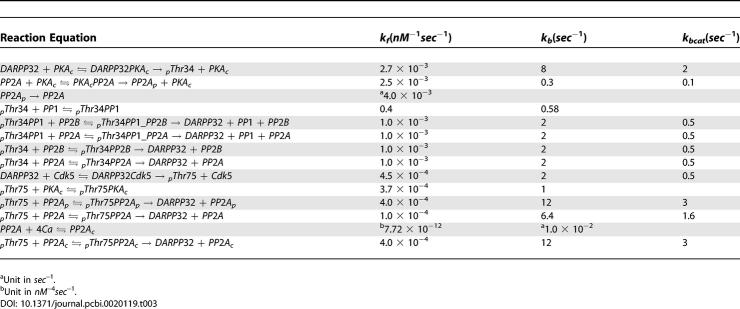
Reactions and Rate Constants of Phosphor/Dephosphorylation of DARPP-32

**Table 4:**
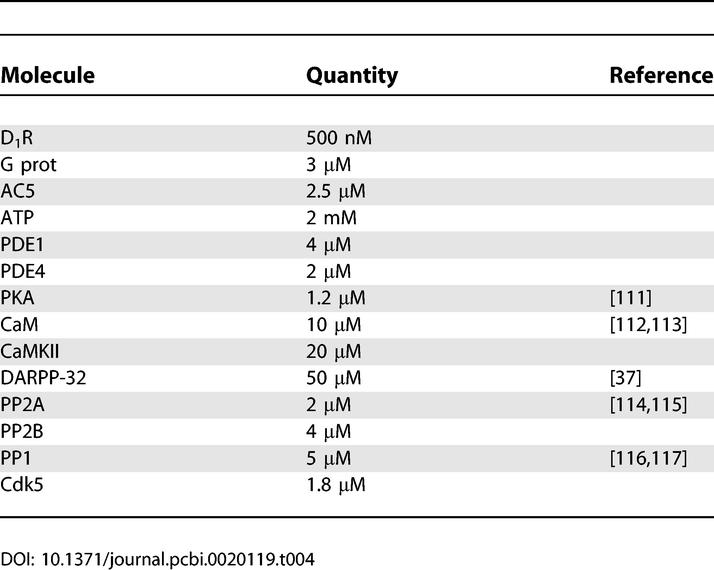
Molecule Quantities

### Dopamine and the G-protein coupled receptor.

The model has a tonic dopamine level of 10 nM, and stimulated or phasic dopamine pulses reach a concentration of 1 μM. The tonic dopamine maintains both the phosphoThr34 and phosphoThr75 at the basal level observed experimentally (via a PP2A-dependent mechanism, see below). In striatum the postsynaptic dopamine D_1_ type receptor (D_1_R) is coupled to the G_olf_ type of GTP-binding protein [84]. The D_1_R can bind to either the inactive G protein first, and then dopamine, or dopamine first and then the inactive G protein [85–87]. Once the complex is formed, it rapidly dissociates into ligand-receptor complex, inactive G_βγ_ subunit, and the active G_olfα_GTP. G_olfα_GTP binds to adenylate cyclase (see below), and also autohydrolyzes into G_olfα_GDP which then binds to G_βγ_ to regenerate inactive G protein. Compared to G_sα_, G_olfα_ binds to AC with a higher K_d_ and has a faster rate of hydrolysis [88].

### Cyclic AMP formation and PKA activation.

Active G_olfα_ binds to and activates adenylate cyclase type V (AC5) in the striatum [70,71,87], which produces cyclic AMP (cAMP). A simplified model of the AC5 enzyme reaction was created by combining several intermediate steps from a detailed AC model [89]. The rate constants for the simplified AC5 model were adjusted, using the nonlinear least-squares regression algorithm of Dynafit [90] (http://www.biokin.com/dynafit/). Figure S3 shows that the time course of cAMP production is almost identical to that of the Dessaur model for our conditions [89].

Calcium causes a 50% reduction in the activity of AC5 [91], though it is not known whether calcium binds to AC5 before or after binding to G_olfα_. In the model, calcium is allowed to bind to the inactive AC5, which then can be activated by G_olfα_GTP. The results do not differ if calcium also is allowed to bind to active AC5. A step of regeneration of ATP is included that allows for a steady-state concentration of ATP in the absence of stimulation. ATP is only modeled explicitly in the formation of cAMP and not in any other enzymatic phosphorylation step below where it is not assumed to be a rate-limiting substrate.

Several types of phosphodiesterases (PDE) degrade cAMP in the striatum. The model includes the calcium-activated PDE1 [92] and a constitutively active form (referred to as PDE4) that represents PDE4B, PDE10A, and PDE7 [93–96].

cAMP binds to and activates PKA by binding to the regulatory subunit of PKA, releasing two catalytic subunits (PKAc). The regulatory subunit has two binding sites for cAMP, one with high affinity and one with low affinity [97]. These parameters were adjusted in the model to reproduce the activation curve for the holoenzyme [98,99] and the activation rate observed using Fluorescence Resonance Energy Transfer imaging [100].

### Glutamate and calcium-activated enzymes.

Glutamate is modeled by its effect on calcium; thus, glutamate is simulated as a calcium elevation. Calcium binds to CaM, which has four calcium-binding sites: two N sites (fast, high affinity), and two C sites (slow, low affinity). Calcium binds to CaM in pairs, producing the active Ca_4_CaM in two consecutive steps. The rate constants are obtained from [101,102]. Ca_4_CaM binds to and activates several molecules in the striatum. The rate constants for PDE1 activation were adjusted to produce 50% activation of PDE1 when model calcium concentration is 400 nM [92,103]. PP2B has a very high affinity for Ca_4_CaM [104,105]. PP2B has a lower affinity for CaM than for Ca_4_CaM, and the calcium dissociation rate of PP2B-CaM and PP2B-Ca_2_CaM is slower than for PP2B-Ca_4_CaM [106].

CaMKII was included in the model as a buffer for CaM; therefore only three reactions are included: reversible binding of CaM to CaMKII; a slow autophosphorylation into CaMKIIp, in which CaM is trapped; and dephosphorylation of CaMKIIp by PP1. Details of autophosphorylation, e.g., [68], were specifically excluded.

### Phosphorylation of DARPP-32.

DARPP-32 activity is regulated by phosphorylation on two sites: Thr34 and Thr75. The catalytic subunit of PKA catalyses phosphorylation of DARPP-32 at Thr34 [73,107]; Cdk5 catalyses phosphorylation of DARPP-32 at Thr75 [15]. Little is known about the regulation of Cdk5, but experiments show that there is a basal level of phosphorylation at Thr75 due to Cdk5 activity [108]. Thus, a constant amount of active Cdk5 is included in the model, to produce the level of phosphoThr75 (25%) that has been experimentally measured [15]. It is known that phosphorylation of one site can affect phosphorylation rates on other sites of the molecule [72,73]; it therefore seems unlikely that the phosphorylation of Thr34 is independent of phosphorylation of Thr75 [15]. When these sites are assumed independent, allowing simultaneous phosphorylation at Thr34 and Thr75 on the same individual molecule, the results are not compatible with experimental data (unpublished data). Thus, for simplicity, simultaneous phosphorylation on both Thr34 and Thr75 are excluded. We have also chosen not to include phosphorylation at Ser102 and Ser137 since these sites are not known to be directly regulated by dopamine or glutamate.

### Dephosphorylation of DARPP-32 and regulation of PP2A.

Thr34 is dephosphorylated by PP2B when this phosphatase is bound to Ca_4_CaM [16]; in contrast, Thr75 is dephosphorylated by PP2A [65]. Though PP2A is constitutively active, its activity is enhanced by phosphorylation [25] and calcium [17]. Therefore, the model has three active forms of PP2A: the basal form with a low k_cat_, a phosphorylated form (PP2Ap, phosphorylated by PKA) with a higher k_cat_ [25], and a form that is activated directly by calcium, PP2Ac. Lacking experimental measurements of specific activity of calcium-activated PP2A, the activity of PP2Ac is set the same as that for phosphorylated PP2A. Furthermore, the calcium-dependent dephosphorylation of Thr34 (by PP2B) and Thr75 (by PP2A) are balanced to give approximately equal relative dephosphorylation levels as well as dephosphorylation dynamics following moderate increases of calcium as in [17,27].

### Activity of phosphorylated DARPP-32.

When DARPP-32 is phosphorylated at Thr75, it binds to and inactivates PKA [15] with an affinity of 2.7 μM [15]. When DARPP-32 is phosphorylated at Thr34 it binds to and inactivates PP1, with an affinity of 1 nM [14]. Since the Thr34 phosphorylated site of DARPP-32 binds to the active zone of PP1 [109], the rate of dephosphorylation of phosphoThr34 by PP2B is probably decreased when bound to PP1. Nonetheless, the very high affinity of phosphoThr34 for PP1 implies that almost all phosphoThr34 is bound to PP1, and if Thr34 can be dephosphorylated only when unbound, phosphoThr34 increases to the amount of PP1*.* This result is contrary to experimental data which reveals that the amount of DARPP-32 phosphorylated on Thr34 is less than 1% [27,110]. Thus the model uses the same dephosphorylation rates for PP1 bound and unbound phosphoThr34 to reproduce experimental measurements. No qualitative changes in the results are seen if the dephosphorylation rate is decreased 50% when phosphoThr34 is bound to PP1. In contrast, due to the much lower affinity of phosphoThr75 for PKA as well as the higher amount of phosphoThr75 in the system (basal level 13 μM), results do not change significantly by assuming that phosphoThr75 bound to PKAc can be dephosphorylated by the different PP2A forms. Thus, for simplicity, only unbound phosphoThr75 can be dephosphorylated in the model.

## Supporting Information

Figure S1 Binding of PKAc to phosphoThr75 and PP2A Enzyme Complexes Following a Burst of Transient Calcium and Dopamine Inputs Supplementary information related to Figure 6A–6C and Figure 10. The changes in PKAc-PP2A and PKAc-phosphoThr75 complexes following (A) eight brief, high dopamine pulses, (B) eight brief, high calcium pulses, or (C) eight paired dopamine and calcium pulses while opening different parts of the PKA–PP2A–phosphoThr75 feedback loop. The PKAc complexes increase when dopamine is given alone, but not if a transient calcium input is provided. This decrease in the presence of a rapid calcium input occurs because calcium activates PP2A and thus decreases phosphoThr75. If the reaction (i) and/or (iii) (as indicated in Figure 6E) are opened, PKAc–PP2A and PKAc–phosphoThr75, respectively, stay constant. This explains why in the presence of only dopamine inputs the PKA–PP2A–phosphoThr75 loop works as a shunt and why the addition of calcium allows the loop instead to facilitate enhancement of PKAc.
(821 KB TIF)

Figure S2 Changes in the PKAc:PP1 Ratio (A) Change in the PKAc:PP1 ratio when DARPP-32 concentration is decreased below control. In all cases a combined dopamine and calcium input produce a higher ratio than either input alone.
(B) Effect of calcium dynamics on the PKAc:PP1 ratio. Complementary information to the results shown in Figure 5D. Eight fast, high calcium pulses increase the PKAc:PP1 ratio much more than eight slower and lower calcium pulses (as in Figure 4B, dashed lines). The red trace is the control trace from Figure 5D when brief dopamine and calcium inputs are paired. A steady-state calcium elevation which is large (2,000 nM, dashed line) or moderate (300 nM, dotted line) instead decreases the PKAc:PP1 ratio since cAMP production is decreased and PP2B-dependent dephosphorylation of phosphoThr34 is enhanced.
(599 KB TIF)

Figure S3 Comparison between the Simplified, Reduced AC5 Model (Solid Line) and the Original Full Model (Dashed Line) by Dessauer et al. The formation of cAMP (red) from 2 mM ATP and the reduction of the free form of AC5 enzyme (blue) show similar dynamics in both versions of the model.
(317 KB TIF)

## Abbreviations

AC5: adenylate cyclase type V
CaM: calmodulin
cAMP: cyclic AMP
cdk5: cyclin-dependent kinase 5
ISI: interstimulus interval
LTP: long-term potentiation
PDE: phosphodiesterases
PKA: protein kinase A
PKAc: catalytic subunit of PKA
PP1: protein phosphatase 1
PP2A: protein phosphatase 2A
PP2B: protein phosphatase 2B
Thr34: Threonine 34
Thr75: Threonine 75
